# Utilizing Post‐Mortem Brain DNA Methylomic Data to Conduct an Epigenome‐Wide Association Study of *APOE* Genotype

**DOI:** 10.1002/alz70855_097108

**Published:** 2025-12-23

**Authors:** Luke Weymouth, Rebecca G. Smith, Adam R. Smith, Jonathan Mill, Aaron Jeffries, Ehsan Pishva, Katie Lunnon

**Affiliations:** ^1^ University of Exeter, Exeter, United Kingdom; ^2^ Maastricht University, Maastricht, Netherlands

## Abstract

**Background:**

The *APOE* ε4 polymorphism is the most well‐established genetic risk factor for sporadic Alzheimer's disease (AD). Although the underlying mechanisms remain unclear, AD has well‐documented environmental and genetic risk factors, suggesting an interplay that may involve epigenetic processes such as DNA methylation. We propose that DNA methylation occurs in an *APOE* allele‐specific manner, serving as a pathway through which genotype drives downstream physiological changes and disease development.

**Methods:**

This research utilized pre‐existing data from over 2,300 extensively characterized pre‐frontal cortex post‐mortem brain samples, collected from various cohorts (as described in PMID: 34112773). *APOE* genotype for each sample was either directly assessed or imputed from genotyping data generated using the Infinium Global Screening Array. DNA methylation profiles were obtained using Illumina 450K or EPIC arrays, with consistent, stringent quality control and data normalization procedures implemented to ensure consistency and reliability. An epigenome‐wide association study was conducted to examine the influence of *APOE* genotype on genome‐wide DNA methylation patterns, accounting for AD pathology and other confounders including age, sex, post‐mortem interval and cell proportions.

**Results:**

Our findings reveal distinct DNA methylation differences among *APOE* allele groups, suggesting a possible epigenetic mechanism through which *APOE* ε4 influences gene regulation.

**Conclusion:**

This extensive analysis underscores the role of DNA methylation as a potential mediator of genetic vulnerability to AD in the context of *APOE*, supporting further exploration into its contribution to disease progression.

